# Supplementary material for: Supercritical density fluctuations and structural heterogeneity in supercooled water-glycerol microdroplets
Source: Nat Commun. 2024 Dec 5;15:10610. doi: 10.1038/s41467-024-54890-y (PMC11621323; doi:10.1038/s41467-024-54890-y)
Supplement: Supplementary file 1 — Supplementary Information [file 41467_2024_54890_MOESM1_ESM.pdf]

Supplementary information:
Supercritical density fluctuations and structural heterogeneity in supercooled water-glycerol microdroplets

Sharon Berkowicz[1,*], Iasonas Andronis[1,*], Anita Girelli[1], Mariia Filianina[1], Maddalena Bin[1], Kyeongmin Nam[2], Myeongsik Shin[2], Markus Kowalewski[1], Tetsuo Katayama[3,4], Nicolas Giovambattista[5,6], Kyung Hwan Kim[2], and Fivos Perakis[1,†]

[1]Department of Physics, AlbaNova University Center, Stockholm University, SE-10691 Stockholm, Sweden
[2]Department of Chemistry, Pohang University of Science and Technology (POSTECH), Pohang 37673, Republic of Korea
[3]Japan Synchrotron Radiation Research Institute, Kouto 1-1-1, Sayo, Hyogo 679-5198, Japan
[4]RIKEN SPring-8 Center, Kouto 1-1-1, Sayo, Hyogo 679-5148, Japan
[5]Department of Physics, Brooklyn College of the City University of New York, Brooklyn, NY 11210, USA.
[6]The Graduate Center of the City University of New York, New York, NY 10016, USA.
[*]These authors contributed equally.
[†]Email: f.perakis@fysik.su.se

Supplementary Note 1 The structure factor for a multi-component molecular liquid

The coherent scattering intensity $I(\mathbf{q})$ for a multi-molecular liquid can be written as [1]

$$I(\mathbf{q}) = \sum_{n}^{N}\sum_{m}^{N} f_n(\mathbf{q}) f_m(\mathbf{q}) e^{i\mathbf{q}\cdot(\mathbf{r}_n - \mathbf{r}_m)}, \tag{S1}$$

where $f_n(\mathbf{q})$ is the molecular form factor, $\mathbf{r}_n$ is the center of mass position of molecule n, and N is the number of molecules. Separation of the above expression into terms $n = m$ (self-scattering term, I_{self}) and $n \neq m$ (cross-term, I_{cross}) yields

$$I(\mathbf{q}) = \sum_{n}^{N} f_n^2(\mathbf{q}) + \sum_{n}^{N}\sum_{m\neq n}^{N} f_n(\mathbf{q}) f_m(\mathbf{q}) e^{i\mathbf{q}\cdot(\mathbf{r}_n - \mathbf{r}_m)} = N I_{self}(\mathbf{q}) + I_{cross}(\mathbf{q}). \tag{S2}$$

In addition, one can rewrite the self-term as a sum of self-terms for each molecular species j, summing over the total number of species J in the solution:

$$N I_{self}(q) = \sum_{j}^{J} N_j f_j^2(q) = N \sum_{j}^{J} x_j f_j^2(q), \tag{S3}$$

where N_j is the number of molecules of species j and $x_j = N_j/N$ is the corresponding molar fraction. Note that averaging over the azimuthal angle allows to drop the vector notation. Specifically, in the case of glycerol-water solution we have

$$I_{self}(q) = x_w f_w^2(q) + x_g f_g^2(q), \tag{S4}$$

where the subscripts w and g denote water and glycerol, respectively.

The structure factor $S(q)$ is defined as the coherently scattered intensity per molecule normalized by the self-scattering intensity of the independent molecules [1], i.e.

$$S(q) = \frac{I(q)}{N I_{self}(q)} = 1 + \frac{I_{cross}(q)}{N I_{self}(q)}, \tag{S5}$$

which oscillates around $S(q) = 1$. The structure factor is then calculated from the experimental data as

$$S(q) = \frac{\alpha I_{corr}(q) - I_{inc}(q)}{I_{self}(q)}, \tag{S6}$$

where $I_{corr}(q)$ is the corrected total X-ray scattering and $I_{inc}(q) = x_w I_{inc,w}(q) + x_g I_{inc,g}(q)$ is the incoherent (Compton) scattering intensity from glycerol-water solution. The coefficient α is introduced to normalize the corrected scattering intensity to electron units (per molecule) and determined by the Krogh-Moe method [2, 3]:

$$\alpha = \frac{\int_{q_1}^{q_2} (I_{self}(q) + I_{inc}(q)) q^2 dq - 2\pi^2 \rho Z^2}{\int_{q_1}^{q_2} I_{corr}(q) q^2 dq}, \tag{S7}$$

where ρ is the average number density of molecules (independent of the species) and $Z = x_w Z_w + x_g Z_g$

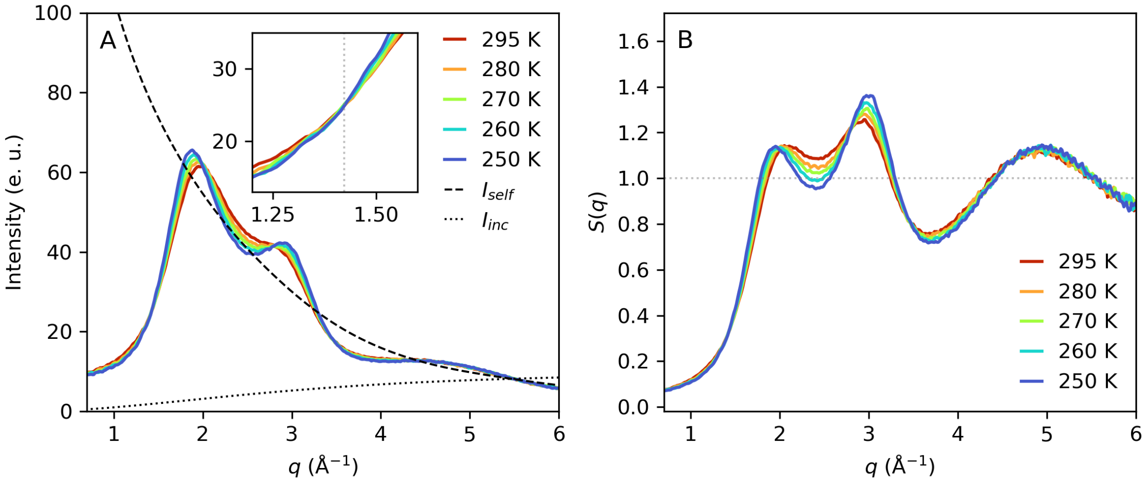

Supplementary Figure. 1 | X-ray diffraction (XRD) measurements of glycerol-water bulk solution ($\chi_g = 3.2\%$ glycerol mole fraction) in a 1-mm thick capillary at different temperatures. (A) The background-subtracted and solid-angle corrected scattering intensity scaled to electron units, i.e. $\alpha I_{corr}(q) - I_{inc}(q)$, using the Krogh-Moe method in Eq. (S7) [2, 3]. $I_{self}(q)$ is the self-scattering term (dashed line) and $I_{inc}(q)$ is the incoherent (Compton) scattering from glycerol-water solution (dotted line). The inset in A shows a zoom-in around the isosbestic point at $q \approx 1.43$ Å^{-1} (gray dotted line) used for normalization of the scattering intensities measured by ultrafast X-ray scattering at SACLA XFEL. (B) The obtained structure factor according to Eq. S6.

is the weighted average number of electrons of water and glycerol. The lower and upper integration limits used are $q_1 = 0.7$ Å^{-1} and $q_2 = 5.9$ Å^{-1}, respectively.

Table-top XRD measurements of glycerol-water bulk solution ($\chi_g = 3.2\%$ glycerol mole fraction) at temperatures $T = 260 - 295$ K are presented in Supplementary Figure 1, where the elastic scattering intensity is scaled to electron units and the structure factors are extracted using Eq. S6-S7.

Supplementary Note 1.1 Scattering intensity corrections

The corrected X-ray scattering $I_{corr}(q)$ was obtained from the measured scattering intensity $I_{meas}(q)$ of supercooled glycerol-water droplets following a number of corrections, including angular integration with solid-angle corrections (using the *Jscatter* software package [4]), sample transmission- and beam polarization corrections and background subtraction. For the scattering intensity of evaporatively cooled droplets recorded at SACLA XFEL, these corrections further included a filtering step to exclude X-ray shots of frozen droplets and missed X-ray shot targets, and averaging of scattering patterns recorded at the same conditions. As background in this case, we utilized the average scattering intensity of the missed X-ray shot targets. To account for small remaining noise (e.g. from variation of X-ray pulse characteristics between shots and exact path of the X-ray beam through the droplet), the background-subtracted average scattering intensities were normalized at the isosbestic point at $q \approx 1.43$ Å^{-1}, as determined by the standard XRD measurements of the same glycerol-water bulk solution ($\chi_g = 3.2\%$ glycerol mole fraction, see inset, Supplementary Figure 1A). In addition, since the scattering patterns measured at SACLA XFEL has a limited q-range ($\sim$ 0.15–1.89 Å^{-1}), we determined the Krogh-Moe coefficient α from the XRD measurements, and scaled the corrected XFEL scattering intensity accordingly to overlap with the XRD intensity at the common temperature of $T \approx 260$ K, as shown in Supplementary Figure 2.

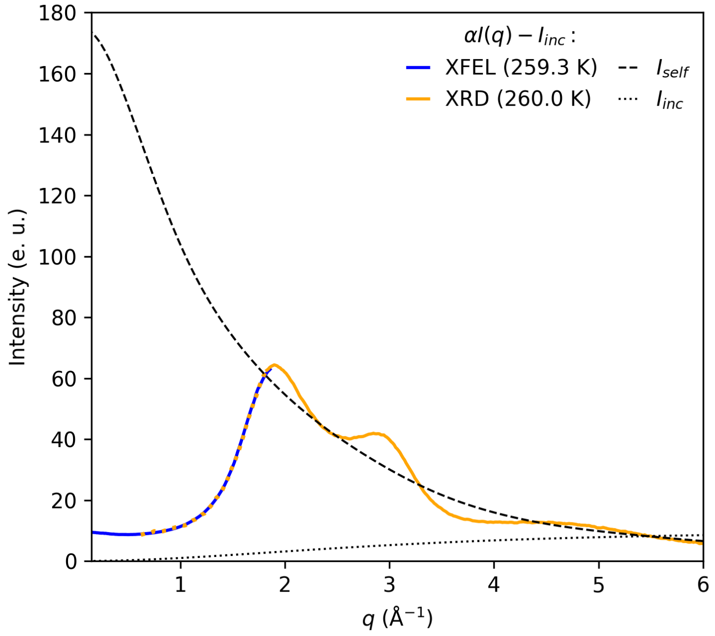

Supplementary Figure. 2 | Comparison of the coherent scattering intensity (in electron units, e.u.) at $T \approx 260$ K. The intensities were measured by ultrafast X-ray scattering at SACLA X-ray free-electron laser (XFEL, blue solid line) and by X-ray diffraction (XRD, orange solid line) with a Mo (K-α) source. The incoherent scattering (dotted line) has been subtracted from the corrected total scattering intensity, i.e. $\alpha I_{corr}(q) - I_{inc}$ where $I_{inc} = x_w I_{inc,w} + x_g I_{inc,g}$ and α is the Krogh-Moe scaling coefficient [2, 3], such that the remaining elastic scattering intensity oscillates around the self-scattering from independent molecules (dashed line), $I_{self} = x_w f_w^2 + x_g f_g^2$.

Supplementary Note 1.2 Molecular form factors and incoherent scattering intensity for glycerol and water

For the individual contributions of water and glycerol to the concentration-weighted molecular form factor (Eq. (S4)) and incoherent scattering for the glycerol-water mixture, we have used the following approximations: (i) for water, we used the molecular form factor f_w and incoherent scattering intensity $I_{inc,w}$ from quantum chemical calculations in Ref. [5], as utilized in a previous experiment on pure water [6]. (ii) For glycerol, however, we calculated the molecular form factor f_g from the average energy-minimized conformation for a single glycerol molecule using the Debye-scattering formula [1]

$$|f(q)|^2 = \sum_n \sum_m f_n^a(q) f_m^a(q) \frac{\sin(q r_{nm})}{q r_{nm}}, \tag{S8}$$

which accounts for orientational averaging. Here, f refers to the molecular form factor as above while f_n^a refers to the atomic form factor for atom n; r_{nm} is the distance between atom n and m. In addition, we approximated the incoherent scattering intensity from glycerol $I_{inc,g}$ as a sum of the independent atomic contributions, i.e.

$$I_{inc} = \sum_n I_{inc,n}^a, \tag{S9}$$

where $I_{inc,n}^a$ is the incoherent scattering intensity from atom n. For the atomic form factors as well as for the atomic incoherent scattering intensities, we utilized tabulated values from Ref. [7].

Supplementary Note 1.3 Calculation of the structure factor from the radial distribution function

The scattering intensity of a liquid (Eq. S2) arising from short-range order can be expressed in terms of the deviation of the local number density $\rho(\mathbf{r})$ from the average density $\bar{\rho}$ [1] as follows

$$I(\mathbf{q}) = NI_{self}(\mathbf{q}) + \sum_{n}^{N} \int_V f_n(\mathbf{q}) f_m(\mathbf{q}) [\rho(\mathbf{r}_{nm}) - \bar{\rho}] e^{i\mathbf{q}\cdot(\mathbf{r_n}-\mathbf{r_m})} dV_m \ , \tag{S10}$$

where $\rho(\mathbf{r}_{nm})$ refers to the local number density in volume-element dV_m at position $\mathbf{r}_m$ with respect to the reference position $\mathbf{r}_n$. Averaging over reference positions yields

$$I(\mathbf{q}) = NI_{self}(\mathbf{q}) + N\langle f(\mathbf{q})\rangle^2 \int_V [\rho(\mathbf{r}) - \bar{\rho}] e^{i\mathbf{q}\cdot\mathbf{r}} dV \ , \tag{S11}$$

where we assumed an average scattering amplitude for each pair of molecules (n and m), i.e. $f_n(\mathbf{q}) f_m(\mathbf{q}) \simeq \langle f(\mathbf{q})\rangle^2$. Further averaging over the azimuthal angle then yields

$$I(q) = NI_{self}(q) + N\langle f(q)\rangle^2 \int_0^\infty \bar{\rho}[g(r) - 1] \frac{\sin(qr)}{qr} 4\pi r^2 \, dr \ . \tag{S12}$$

where $g(r) = \rho(r)/\bar{\rho}$ is the radial distribution function.

In the dilute limit we can assume that $\langle f(q)\rangle^2 = (\sum_j^J x_j f_j)^2 \simeq \sum_j^J x_j f_j^2(q) = I_{self}$. Thus, according to Eq. S5, the structure factor describing the short-range order of a dilute or single-component solution can be expressed as [1]

$$S(q) \simeq 1 + 4\pi\bar{\rho} \int_0^\infty r[g(r) - 1] \frac{\sin(qr)}{q} \, dr \ . \tag{S13}$$

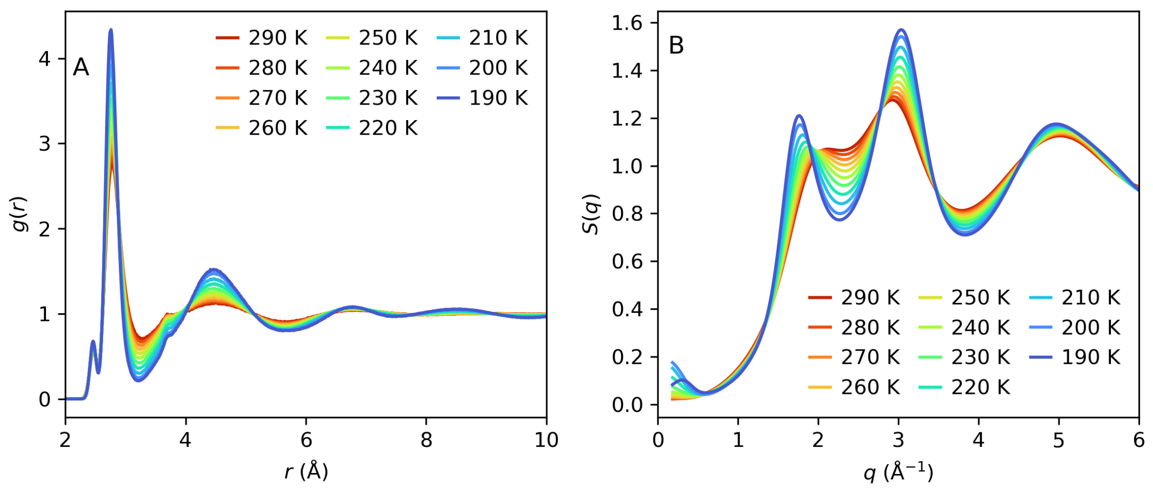

Supplementary Figure. 3 | Molecular dynamics (MD) simulations of glycerol-water solution (χ_g = 3.2% glycerol mole fraction) at different temperatures. (A) The radial distribution function $g(r)$ versus the radial distance r. The $g(r)$ is calculated by considering all the heavy atoms (O and C) in the solution and excluding the H atoms. (B) The structure factor $S(q)$ calculated from the $g(r)$ shown in panel (A), according to Eq. S13.

Supplementary Note 1.4 Reproducibility and control of experimental conditions

The reproducibility of the measurements can be confirmed by the data obtained during two independent runs, illustrated in Fig. S4. During run 1, we were able to reach temperatures from 259.8 K down to 232.1K, whereas in run 2 we could access the range from 259.3 K down to 229.3 K. We observe that the data is highly reproducible within the experimental error bars as can be seen by the comparison in the $S(q)$ line shape (Fig. S4A and B), but also from the temperature dependence of the q_1 peak position and isothermal compressibility κ_T (Fig. S4C and D). As an additional independent experimental control, we have also included data collected with a tabletop X-ray diffractometer (empty circles in Fig. S4C), which aligns well with the XFEL data. This consistency among various independent measurements validates our results and indicates that the observed trends are independent of changes in the experimental configuration.

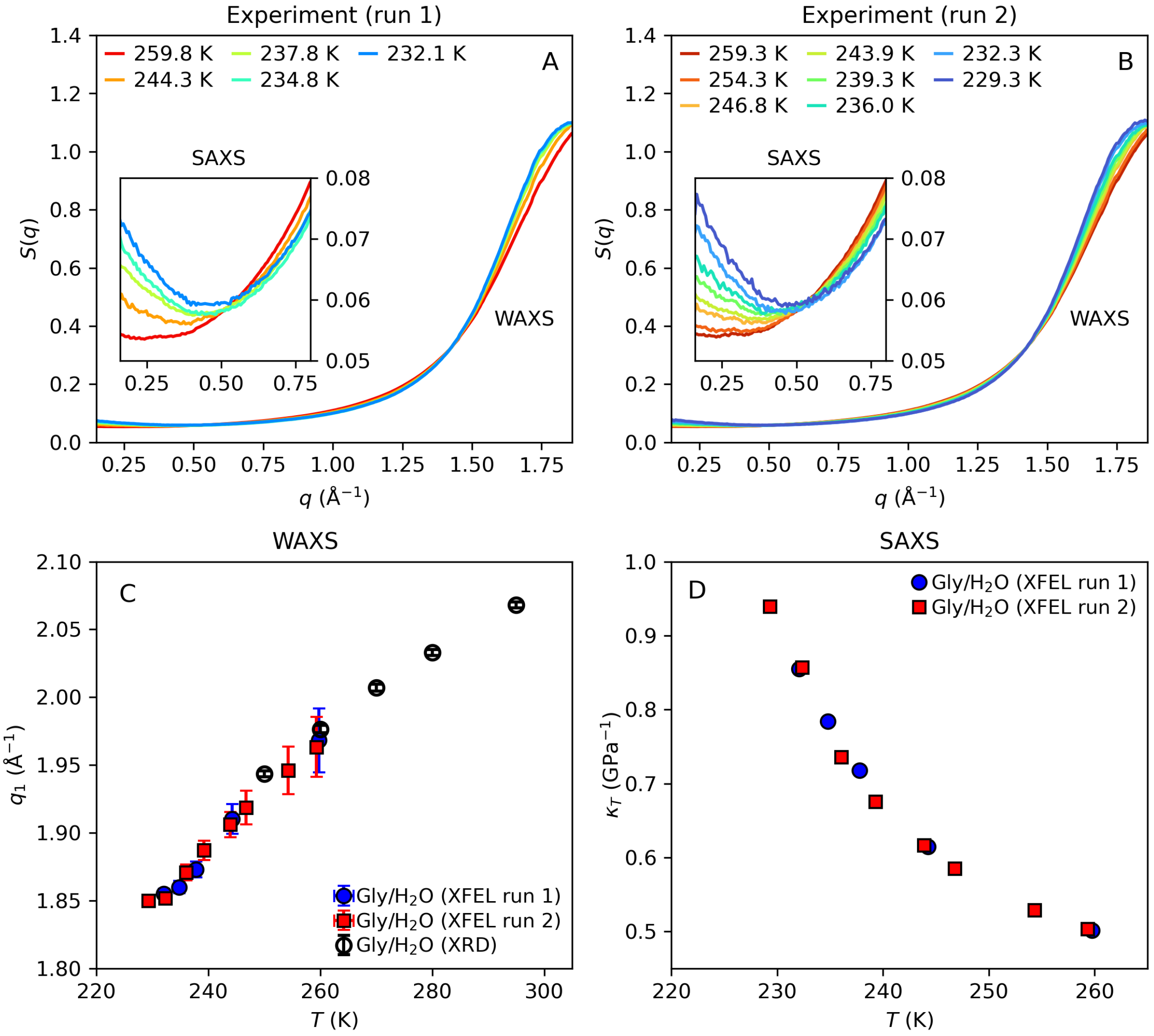

Supplementary Figure. 4 | Reproducibility and control of experimental results. (A-B) The structure factor $S(q)$ obtained during two independent measurements (run 1 and run 2). (C-D) The corresponding $S(q)$ peak position, q_1, and isothermal compressibility, κ_T obtained for the two runs. Errorbars indicate the standard error.

Supplementary Note 2 Experimental small-angle X-ray scattering analysis

The small-angle X-ray scattering structure factor is presented in Supplementary Figure 5A. The structure factor was obtained from the glycerol-water microdroplets ($\chi_g = 3.2\%$ glycerol mole fraction) at SACLA XFEL. The structure factor S_{tot} is decomposed as

$$S_{tot}(q) = S_N(q) + S_A(q) \tag{S14}$$

where S_N and S_A are the normal and anomalous components, respectively, of the structure factor (see main manuscript).

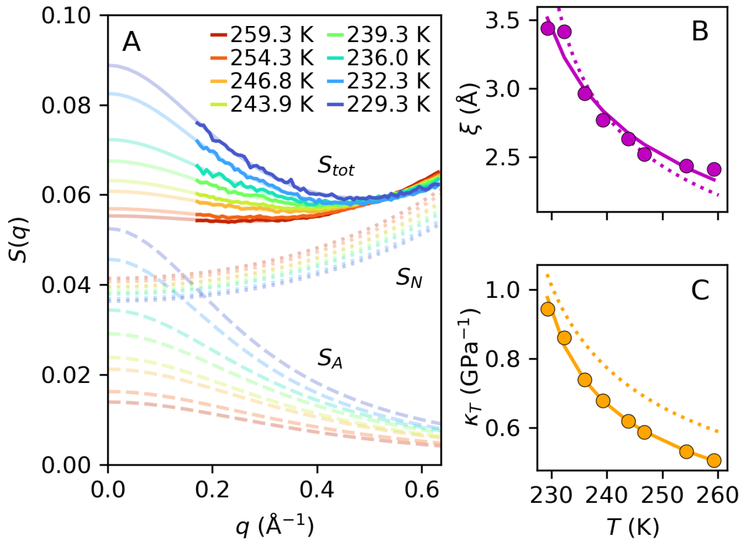

Supplementary Figure. 5 | Analysis of the small-angle X-ray scattering (SAXS) of glycerol-water microdroplets ($\chi_g = 3.2\%$ glycerol mole fraction) obtained at SACLA XFEL. (A) Decomposition of the total SAXS structure factor (S_{tot}) into normal (S_N, dotted lines) and anomalous (S_A, dashed lines) components. The experimental data for different temperatures is shown as full-colored solid lines while shaded solid lines denote the fits according to Eq.S14 (see also Methods in the main manuscript). Subpanels (B) and (C) show the correlation length ξ and isothermal compressibility κ_T extracted from the fits in A. The lines are power law fits to the correlation length and isothermal compressibility according to Eq. (2) in the main manuscript for glycerol-water solution (solid lines) and pure water (dashed lines) from Ref. [8].

Supplementary Note 3 Droplet temperature estimation

The Knudsen evaporation model employed in this work for the estimation of the droplet temperature has been validated in prior studies, including both experimental approaches [6, 9], as well as molecular dynamics (MD) simulations [10]. Given the small deviation observed within independent datasets, we conclude that the temperature of the microdroplet for a given travel time is relatively homogeneous, in agreement with previous studies [6, 9].

We account for the glycerol-water mixture ($\chi_g = 3.2\%$ glycerol mole fraction) by including interpolated experimental thermodynamic parameters of the mixture in the evaporation model. In the Knuden evaporation theory, the evaporation rate Γ, i.e. the rate at which molecules evaporate from the droplet surface, after travel time t is given by

$$\Gamma(t) = \gamma \frac{P^*_{vap}(T_s) A_s(t)}{\sqrt{2\pi m k_B T_s}}. \tag{S15}$$

Here, $P^*_{vap} = P_{vap} - P_0$ is the effective saturation vapor pressure where P_{vap} is the saturation pressure of the mixture and P_0 is the chamber vacuum pressure. $T_s = T_s(t)$ is the current temperature in the droplet surface layer, A_s is the droplet surface area, m is the average molecular mass, and k_B is the Boltzmann constant. Due to the low glycerol concentration, we approximate the evaporation coefficient with $\gamma = 1$ used previously for pure water [10]. The cooling rate on the droplet surface due to evaporation is

$$\frac{dT_s}{dt} = -\Gamma(t) \left[\frac{\Delta H_{vap}(T_s)}{C_p(T_s) \Delta V_s(t) \rho(T_s)} \right], \tag{S16}$$

where ΔH_{vap} is the evaporation enthalpy, C_p is the isobaric heat capacity and ρ is the density of the mixture. V_s is the volume of the droplet surface layer.

As previously done in Refs. [6, 9, 10], the droplet is divided into n spherical shells and the temperature of the droplet is calculated numerically by estimating the heat conduction between shells as a function of time. The heat flow, dQ/dt, between shell n and $n+1$ is calculated using the Fourier's law of thermal conduction

$$\frac{dQ}{dt} = -\frac{4\pi r_n^2 \kappa(T_n)}{\Delta r(t)} \left[T_{n+1} - T_n \right], \tag{S17}$$

where κ is the thermal conductivity of the mixture, Δr is the shell thickness; r_n and T_n are the outer radius and the current temperature of shell n, respectively. The temperature change due to heat flow between shells is then given by

$$dT_n = \frac{\Delta Q}{C_p(T_n) M_n}, \tag{S18}$$

where ΔQ is the net heat flowing into shell n, $M_n = \Delta V_n \rho(T_n)$ is the shell mass and ΔV_n is the shell volume.

Due to the large mass difference and the low vapor pressure of glycerol compared to water, we assume that only water molecules evaporate. During the numerical calculation [6, 9, 10], the droplet radius r is updated after each iteration (i.e, time step dt) by accounting for the droplet volume change due to the number of evaporated water molecules n_{evap}:

$$\frac{r^3(t)}{r_0^3} = \frac{V(t)}{V_0} = \frac{V_0 - n_{evap}(t) V_m}{V_0} \tag{S19}$$

where $n_{evap}(t) = \Gamma(t)dt$ and V is the droplet volume. V_m is the molar volume of water in the mixture, which is approximated by the molar volume in pure water. In addition, after each iteration, we account for the slightly increased glycerol molar fraction x_g

$$x_g(t) = \frac{n_g}{n_g + (n_w - n_{evap}(t))}, \tag{S20}$$

where n_g and n_w are the initial number of molecules of glycerol and water, respectively.

The average droplet temperature $T(t)$ is calculated by averaging the temperatures $\{T_n\}$ of the shells. The resulting estimated droplet temperatures and glycerol concentrations are presented in Supplementary Figure 7F. The droplet travel time t in the vacuum chamber is calculated from the known travelled droplet distance z from the liquid jet nozzle, the droplet frequency f and the droplet-droplet distance l_{dd}, as

$$t = \frac{z}{f \cdot l_{dd}}. \tag{S21}$$

Both the initial droplet radius r_0 and the droplet-droplet distance l_{dd} are calibrated from *in situ* optical microscope images. The thermodynamic properties and the input parameters used for the temperature estimation are summarized in Supplementary Figure 7A-E and Table 1, respectively.

We estimate that the main source of experimental uncertainty in the droplet temperature estimation by Knudsen theory is likely the droplet size. This uncertainty arises from the size determination from 2D microscope images, where the cross-section of the 3D droplets depends on the camera sharpness and focusing. Based on the microscope images, we estimate that the uncertainty in the droplet diameter is, at most, σ_d=±3 μm, where d_0=18.7 μm is the determined droplet diameter. Such deviations in droplet size would result in different droplet temperatures calculated with Knudsen evaporation theory.

The resulting uncertainty in the temperature decreases upon cooling, from approximately ≈3 K at $T_0 = 260\ K$ to ≈1 K at $T_0 = 230\ K$ (Fig. S6). Therefore, we conclude that the uncertainty in droplet size and temperature should not significantly alter the observed temperature trends of the experimental q_1 position and compressibility κ_T. Furthermore, the high level of data reproducibility suggests that the underlying physical processes influencing the droplet behavior remain robust despite any minor variations in droplet temperature.

Supplementary Table 1 | Droplet temperature estimation parameters

Glycerol molar (mass) fraction	x_g (w_g)	3.2% (14.5 wt%)
Droplet diameter	$2r_0$	18.7 μm
Droplet-droplet distance	l_{dd}	85.5 μm
Droplet frequency	f	157 kHz
Travel distances	z	5–65 mm
Initial temperature	T_0	298 K
Chamber vacuum pressure	P_0	1.60 Pa
Time step	dt	1 ns and 10 ns
Number of time steps	N_{steps}	700 000
Number of spherical shells	N_{shells}	100
Evaporation coefficient	γ	1

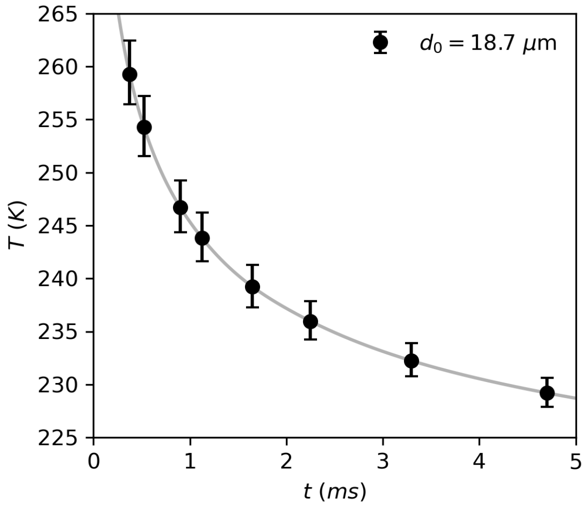

Supplementary Figure. 6 | Droplet temperature estimation calculated with Knudsen evaporation theory for droplets with size $d_0 = 18.7\ \mu m$. The errorbars convey the temperature uncertainty upon cooling, for a droplet size of $\sigma_d = \pm 3\ \mu m$.

Supplementary Note 3.1 Thermodynamic properties of glycerol-water mixtures

Supplementary Note 3.1.1 Density

The density ρ of the glycerol-water mixture is estimated using an empirical formula from Ref. [11]:

$$\rho(T, w_g > 0) = 1 + A(T)\sin(w_g^{1.31}\pi)^{0.81}\left[\rho_w(T) + \frac{\rho_g(T) - \rho_w(T)}{1 + \frac{\rho_g(T)}{\rho_w(T)}(\frac{1}{w_g} - 1)}\right], \tag{S22}$$

where $A(T) = 1.78 \cdot 10^{-6}T^2 - 1.82 \cdot 10^{-4}T + 1.41 \cdot 10^{-2}$, ρ_w is the density of pure water, ρ_g is the density of pure glycerol, w_g is the mass fraction of glycerol. For the density of liquid glycerol we interpolate experimental density data between 156–311 K from Ref. [12], while for water we use an interpolation of experimental density data of hexagonal ice and liquid water from Ref. [13], as used previously [9].

Supplementary Note 3.1.2 Saturation vapor pressure

To estimate the saturation vapor pressure P_{vap} of the glycerol-water mixture utilize an interpolation of experimental data of the concentration-dependent relative vapor pressure P_{rel} of the mixture compared to pure water (at $T = 273.15$ K) from Ref. [14]. Thus,

$$P_{vap}(T, x_g) = P_{vap,w}(T) \cdot P_{rel}(x_g), \tag{S23}$$

where $P_{vap,w}$ is the saturation vapor pressure of water from Ref. [15], as used previously [9].

Supplementary Note 3.1.3 Isobaric heat capacity

For the isobaric heat capacity C_p we use a mass-fraction weighted formula as suggested in Ref. [16],

$$C_p(T, w_g) = \frac{(1 - w_g)C_{p,w}(T)}{1 + aw_g^b} + w_g\left[cB(T) + d\right], \tag{S24}$$

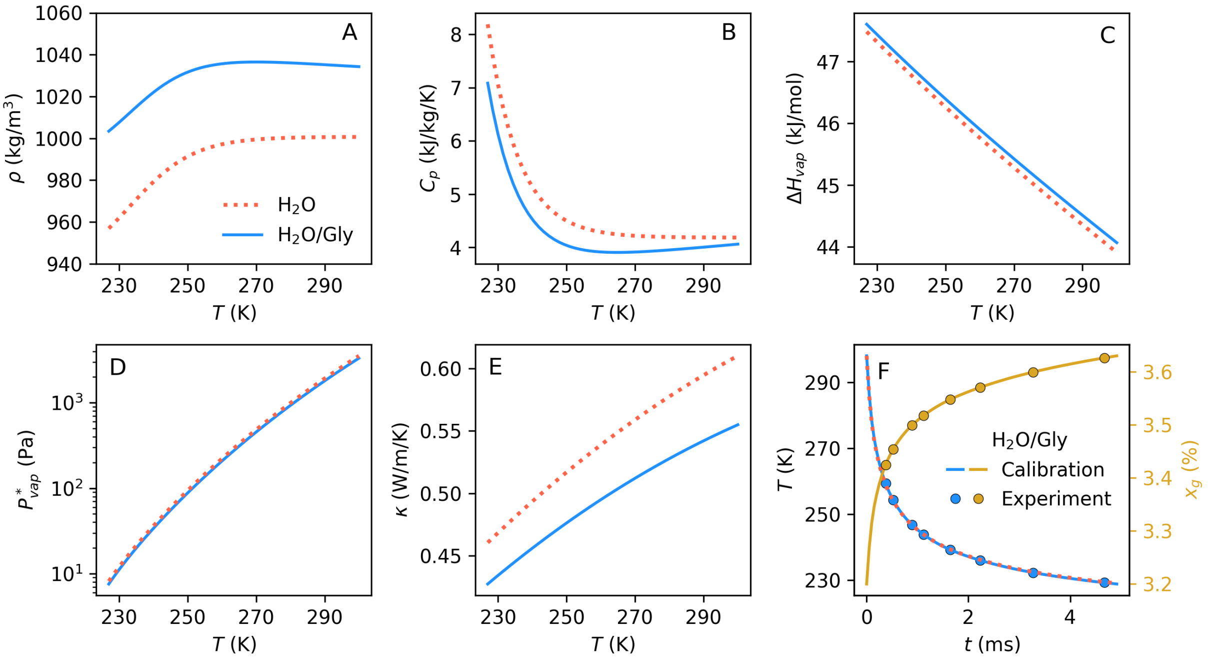

Supplementary Figure. 7 | (A-E) Thermodynamic properties of glycerol-water solution ($\chi_g = 3.2\%$ glycerol mole fraction, blue), compared that of pure water (red), used for the droplet temperature calculations; density ρ, isobaric heat capacity C_p, vaporization enthalpy ΔH_{vap} and effective vapor pressure P^*_{vap} for vacuum chamber pressure $P_0 = 1.6$ Pa, and thermal conductivity κ. (F) The estimated droplet temperature as a function of travel time from the liquid jet nozzle, where curves for pure water and the mixture are nearly overlapping. The slightly increasing glycerol molar fraction x_g versus travel time for the mixture is shown in yellow (right axis).

where $B(T)$ is a baseline (first degree polynomial) determined from a fit to experimental C_p data of 65 wt% glycerol-water mixture, while a, b, c and d are constants obtained by fitting the entire Eq. S24 to experimental data in the range 25–65 wt% glycerol-water from Ref. [14]. As previously [9], for the isobaric heat capacity of pure water $C_{p,w}$ we use an interpolation of experimental data from Ref. [17].

Supplementary Note 3.1.4 Enthalpy of vaporization

To estimate the enthalpy of vaporization H_{vap} for the glycerol-water solution we use a theoretical formula for binary mixtures derived in Ref. [18]:

$$\Delta H_{vap}(T, x_g) = -RT \ln \left[\frac{P_{vap}(T, x_g)}{P_{vap,w}(T)} \right] + \Delta H_{vap,w}(T), \tag{S25}$$

where R is the ideal gas constant and $H_{vap,w}$ is the vaporization enthalpy for pure water from Ref. [19], as used previously [9].

Supplementary Note 3.1.5 Thermal conductivity

The thermal conductivity of the glycerol-water mixture is calculated as a mass-fraction weighted average of the thermal conductivity for pure water κ_w, as previously by interpolating experimental data from Ref. [20], and the linearly temperature-dependent thermal conductivity for pure glycerol κ_g from Ref. [14]:

$$\kappa(T, w_g) = (1 - w_g)\kappa_w(T) + w_g \kappa_g(T) \tag{S26}$$

The accuracy of the above formula was checked against experimental data for glycerol-water mixtures from Ref. [14] providing good agreement for concentrations ranging up to 50 wt% glycerol.

Supplementary Note 4 Compressibility Power law R^2 analysis

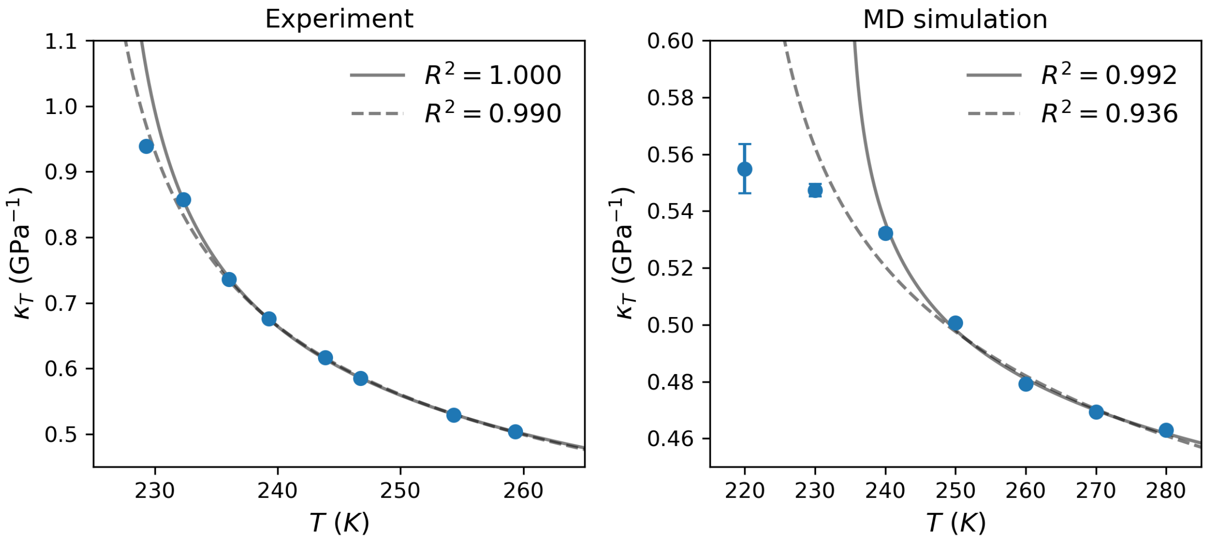

Supplementary Figure. 8 | Comparison of the isothermal compressibility κ_T(T) of glycerol-water obtained from (A) the experiment and (B) the MD simulations. The lines depict power-law fits for different temperature ranges, with (dashed line) and without (solid line) the κ_T at $T = 230K$. Based on the goodness of the fit (R^2 shown in the legend) we observe that both experimental and MD data indicate deviation from the power law behavior at $T = 230K$. Errorbars indicate the standard error.

Supplementary Note 5 MD Simulations Additional Information and Comparisons

The CHARMM36 force field [21, 22] was used to represent the glycerol molecules and the TIP4P/2005 model was used to model the water molecules [23]. The CHARMM force field is a well-known additive, all-atom force field that has been used extensively in the past to study proteins, nucleic acids, lipids, and carbohydrates. The CHARMM force field combined with the TIP4P/2005 water model have been used in the past to study the structural, dynamical, and thermodynamic properties of glycerol-water [24–29]. Our rationale for employing the TIP4P/2005 water model is that it reproduces very well the properties of bulk water and many of the properties of glycerol-water mixtures [26–28]. In particular, the TIP4P/2005 water model reproduces qualitatively well the anomalous properties of water, including the compressibility maximum at 1 bar, and it exhibits a liquid-liquid critical point [30].

Supplementary Note 5.1 Comparison of experimental and MD results

We note that our results from MD simulations, based on the CHARMM36 force field and TIP4P/2005 water model, are in very good agreement with the experiments. To show this we consider a glycerol-water solution with $\chi_g = 3.2\%$ glycerol mole fraction and compare the location of the first peak of the structure factor, q_1, obtained from experiments and MD simulations. As shown in Fig. S9A, the values of q_1 obtained from experiments and MD simulations are in very good agreement with each other, particularly at T>255 K. At lower temperatures, the values of q_1 obtained from MD simulations appear to deviate slightly from the experimental values. A similar trend is observed in the values of q_1 reported from MD simulations of bulk water [30]. Since the q_1 position correlates with the tetrahedrality fraction of water [6], this observation indicates that TIP4P/2005 can underestimate the tetrahedral local coordination at ambient pressure.

The isothermal compressibility κ_T of the glycerol-water solution ($\chi_g = 3.2\%$) calculated from (i) the experimental structure factor measured in the XFEL experiment and (ii) the MD simulations are included in Fig. S9B. As for the case of pure TIP4P/2005 water, the MD simulations of the glycerol-water mixture reproduce the qualitative increase of κ_T upon cooling. However, our MD simulations underestimate the value of κ_T relative to the experiments. This is not surprising since most empirical rigid/flexible water models, including the TIP4P/2005 model, underestimate the values of κ_T of bulk water at low temperatures [30]. Despite this limitation inherent to most of the available water models, our results from MD simulations in Fig. S9B clearly show a maximum in the κ_T of the glycerol-water solution ($\chi_g = 3.2\%$) at $T \approx 223~K$, at temperatures slightly below the lowest temperature accessed in our experiments.

Supplementary Note 5.2 Equilibration check and Production run details

The equilibration time ranged from 200 ns at $T = 290$ K to 2 μs at 190 K based on time-dependence of the potential energy shown in Fig. S11. The corresponding simulation time for the production runs was 200 ns and was determined based on the characteristic correlation time obtained from the density-density correlation functions, as shown in Fig. S11. This approach ensures full decorrelation within the production run and allows complete sampling of the density fluctuations at lower temperatures. In addition, we confirm that the mean-square displacement of glycerol and water becomes a linear function of time within the simulation time for the production runs, i.e., both the glycerol and water molecules reach the diffusive regime (see Fig. S11C and Fig. S11D).

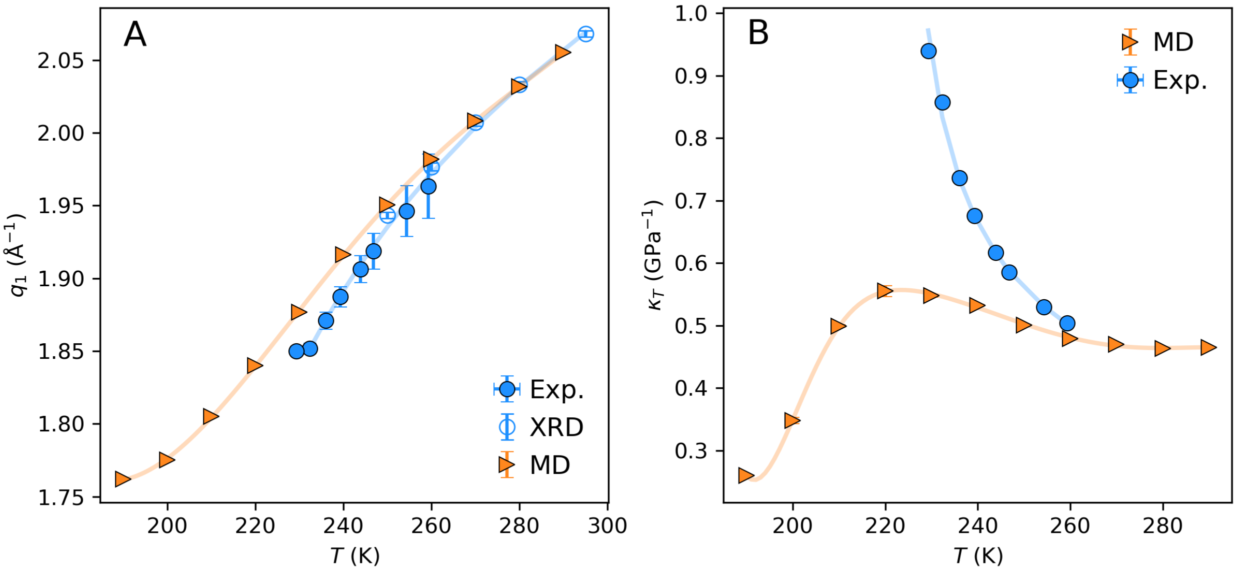

Supplementary Figure. 9 | (A) Comparison of the structure factor first-peak position, $q_1(T)$, obtained from XFEL and XRD experiments (blue filled and empty circles respectively), as well as those from MD simulations (orange triangles) of the studied glycerol-water solution ($\chi_g = 3.2\%$ glycerol mole fraction). The agreement between experiments and MD simulations at $T > 229$ K is remarkably good. (B) Temperature dependence of the isothermal compressibility κ_T of the glycerol-water solution calculated from the experimental structure factor from the XFEL experiment (blue circles) and the MD simulations (orange triangles). As for the case of pure TIP4P/2005 water, the MD simulations of the glycerol-water mixture reproduce the qualitative increase of κ_T upon cooling although they underestimate the value of κ_T relative to the experiments. Note that MD simulations show a clear maxima in the κ_T of the solution at $T \approx 230$ K. Errorbars indicate the standard error.

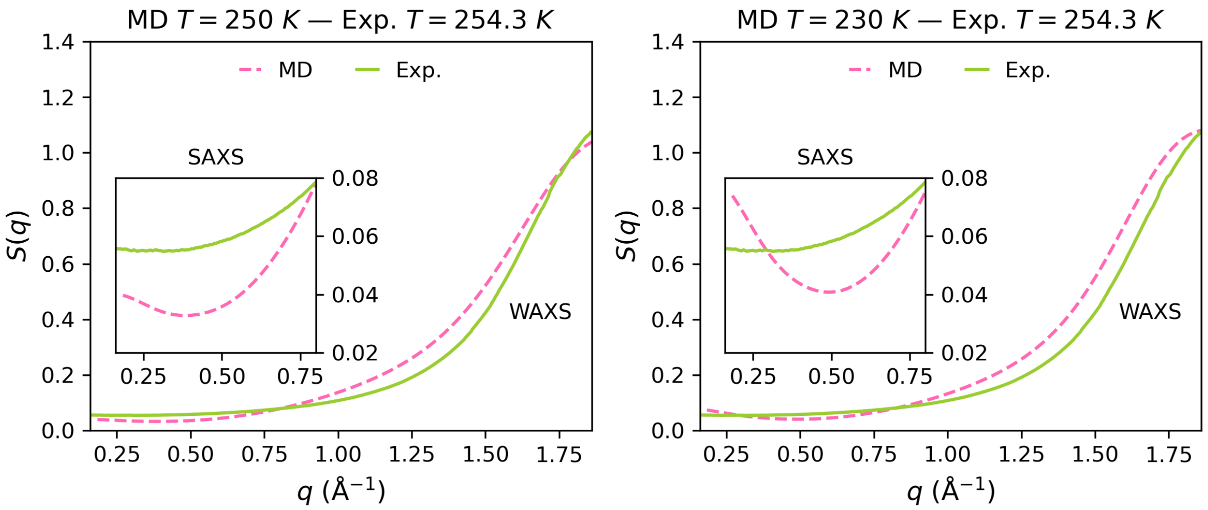

Supplementary Figure. 10 | Direct comparison of the structure factor $S(q)$ obtained from experiment (solid line) and MD simulations (dashed line) for (a) similar temperatures near $T = 250$ K and (b) similar supercooling degrees taking into account the melting point for TIP4P/2005 ($T_m \approx 250$ K at $P = 1$bar).

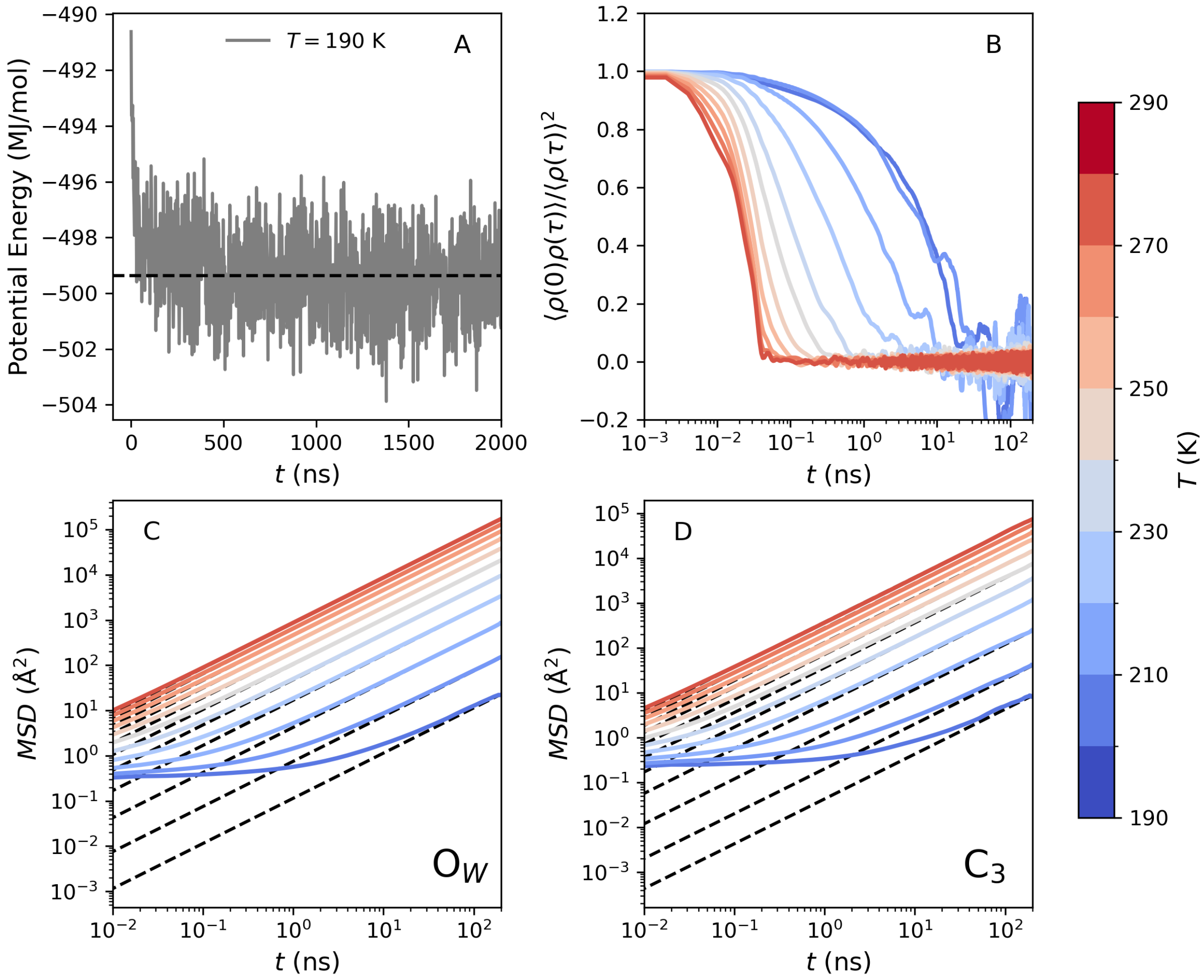

Supplementary Figure. 11 | (A) Potential energy as a function of time during the equilibration of all MD simulations of the glycerol-water solution at T=190K. The horizontal black dashed line represents the average of the last 100 data points and serves as a visual guide. (B) Density-density time correlation of the MD simulations (production runs) of water-glycerol for different temperatures shown in the legend. We observe that full decorrelation occurs within 200 ns, which is the simulation time of the production runs for each temperature. (C) The mean-square displacement of water (with reference to the oxygen, O_W) and (D) that of glycerol (with reference to the central carbon, C_3).

Supplementary Note 5.3 Reproducibility of MD simulations

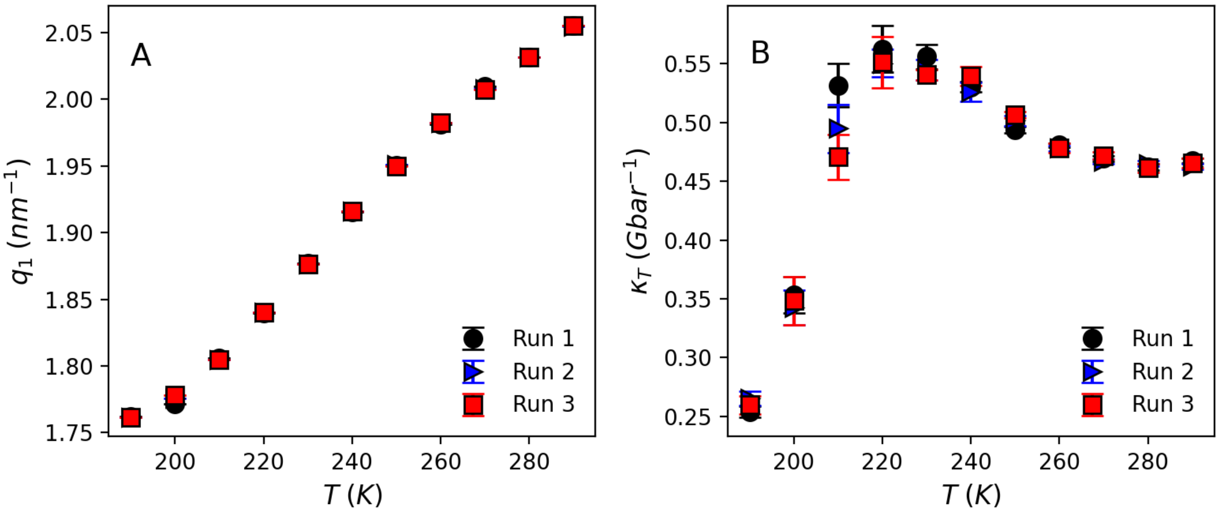

Supplementary Figure. 12 | Results from three MD simulations of a glycerol-water solution at $P = 1$bar ($\chi_g = 3.2\%$); MD simulations start from a different starting configuration. (A) Position of the structure factor first peak, $q_1(T)$, and (B) isothermal compressibility, κ_T(T), as a function of temperature. The values of $q_1(T)$ and κ_T(T) are reproducible among the three runs within the error bars. The MD simulation results reported in the main manuscript are the average values of the three runs shown here. Errorbars indicate the standard error.

Supplementary Note 6 Analysis of solvation layer composition from MD simulations

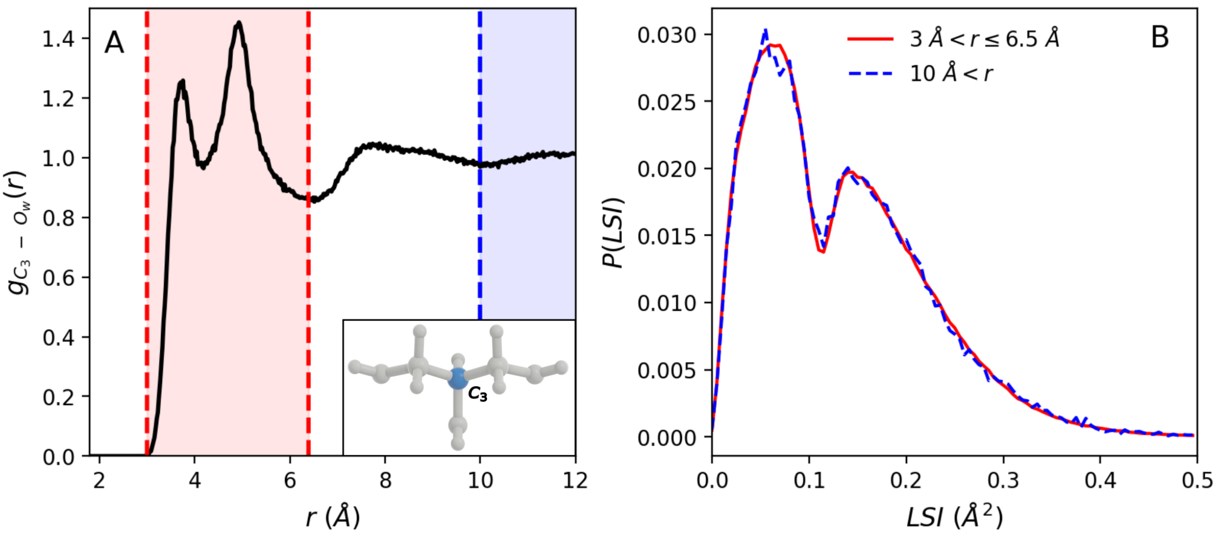

Supplementary Figure. 13 | (A) Site-site partial radial distribution function (RDF) between the central carbon of glycerol (C_3) and the oxygen atoms of water (O_W). The regions corresponding to the first and second hydration shell (3Å$< r <$ 6.5Å) and the bulk region ($r >$ 10Å) are shaded in red and blue, respectively. A representation of a glycerol molecule is included in the inset, with the central carbon highlighted. (B) Comparison of the inherent Local Structure Index (LSI) for water in the first/second hydration shell (solid red line) and in the bulk region (dashed blue line).

References

1. Als-Nielsen, J. & McMorrow, D. in *Elements of Modern X-ray Physics* 1st ed., 113–146 (Chichester, West Sussex, United Kingdom, 2011).
2. Krogh-Moe, J. A method for converting experimental X-ray intensities to an absolute scale. *Acta Cryst.* **9,** 951–953 (1956).
3. Norman, N. The Fourier transform method for normalizing intensities. *Acta Cryst.* **10,** 370–373 (1957).
4. Biehl, R. Jscatter, a program for evaluation and analysis of experimental data. *PLoS One* **14,** e0218789 (2019).
5. Wang, J., Tripathi, A. N. & Smith, V. H. Chemical binding and electron correlation effects in x-ray and high energy electron scattering. *J. Chem. Phys.* **101,** 4842–4854 (1994).
6. Kim, K. H. *et al.* Maxima in the thermodynamic response and correlation functions of deeply supercooled water. *Science* **358,** 1589 (2017).
7. Hubbell, J. H. *et al.* Atomic form factors, incoherent scattering functions, and photon scattering cross sections. *J. Phys. Chem. Ref. Data* **4,** 471–538 (1975).
8. Späh, A. *et al.* Apparent power-law behavior of water's isothermal compressibility and correlation length upon supercooling. *Phys. Chem. Chem. Phys.* **21,** 26–31 (2018).
9. Sellberg, J. A. *et al.* Ultrafast X-ray probing of water structure below the homogeneous ice nucleation temperature. *Nature* **510,** 381 (2014).
10. Schlesinger, D., Sellberg, J. A., Nilsson, A. & Pettersson, L. G. M. Evaporative cooling of microscopic water droplets in vacuo: Molecular dynamics simulations and kinetic gas theory. *J. Chem. Phys.* **144,** 124502 (2016).
11. Volk, A. & Kähler, C. J. Density model for aqueous glycerol solutions. *Exp. Fluids* **59,** 75 (2018).
12. Blazhnov, I. V., Malomuzh, N. P. & Lishchuk, S. V. Temperature dependence of density, thermal expansion coefficient and shear viscosity of supercooled glycerol as a reflection of its structure. *J. Chem. Phys.* **121,** 6435–6441 (2004).
13. Kell, G. S. Density, thermal expansivity, and compressibility of liquid water from 0.deg. to 150.deg.. Correlations and tables for atmospheric pressure and saturation reviewed and expressed on 1968 temperature scale. *J. Chem. Eng. Data* **20,** 97–105 (1975).
14. Soap and Detergent Association. *Physical Properties of Glycerine and its Solutions* (New York, 1963).
15. Murphy, D. M. & Koop, T. Review of the vapour pressures of ice and supercooled water for atmospheric applications. *Q. J. Roy. Meteorol. Soc.* **131,** 1539–1565 (2005).
16. Biddle, J. W., Holten, V. & Anisimov, M. A. Behavior of supercooled aqueous solutions stemming from hidden liquid–liquid transition in water. *J. Chem. Phys.* **141,** 074504 (2014).
17. Angell, C. A., Sichina, W. J. & Oguni, M. Heat capacity of water at extremes of supercooling and superheating. *J. Phys. Chem.* **86,** 998–1002 (1982).
18. Dobruskin, V. K. *Effect of Chemical Composition on Enthalpy of Evaporation and Equilibrium Vapor Pressure* 2010.
19. Somayajulu, G. R. New equations for enthalpy of vaporization from the triple point to the critical point. *Int. J. Thermophys.* **9,** 567–575 (1988).

20. Lide, D. R. *CRC Handbook of Chemistry and Physics* 90th ed. (Boca Raton, 2010).

21. Vanommeslaeghe, K. *et al.* CHARMM general force field: A force field for drug-like molecules compatible with the CHARMM all-atom additive biological force fields. *J. Comput. Chem.* **31,** 671–690 (2010).

22. Reiling, S., Schlenkrich, M. & Brickmann, J. Force field parameters for carbohydrates. *J. Comput. Chem.* **17,** 450–468 (1996).

23. Abascal, J. L. F. & Vega, C. A general purpose model for the condensed phases of water: TIP4P/2005. *J. Chem. Phys.* **123,** 234505 (2005).

24. Egorov, A. V., Lyubartsev, A. P. & Laaksonen, A. Molecular Dynamics Simulation Study of Glycerol–Water Liquid Mixtures. *J. Phys. Chem. B* **115,** 14572–14581 (2011).

25. Dashnau, J. L., Nucci, N. V., Sharp, K. A. & Vanderkooi, J. M. Hydrogen Bonding and the Cryoprotective Properties of Glycerol/Water Mixtures. *J. Phys. Chem. B* **110,** 13670–13677 (2006).

26. Jahn, D. A., Wong, J., Bachler, J., Loerting, T. & Giovambattista, N. Glass polymorphism in glycerol–water mixtures: I. A computer simulation study. *Phys. Chem. Chem. Phys.* **18,** 11042–11057 (2016).

27. Jahn, D. A., Akinkunmi, F. O. & Giovambattista, N. Effects of Temperature on the Properties of Glycerol: A Computer Simulation Study of Five Different Force Fields. *J. Phys. Chem. B* **118,** 11284–11294 (2014).

28. Akinkunmi, F. O., Jahn, D. A. & Giovambattista, N. Effects of Temperature on the Thermodynamic and Dynamical Properties of Glycerol–Water Mixtures: A Computer Simulation Study of Three Different Force Fields. *J. Phys. Chem. B* **119,** 6250–6261 (2015).

29. Daschakraborty, S. How do glycerol and dimethyl sulphoxide affect local tetrahedral structure of water around a nonpolar solute at low temperature? Importance of preferential interaction. *J. Chem. Phys.* **148,** 134501 (2018).

30. Pathak, H. *et al.* Temperature dependent anomalous fluctuations in water: shift of $\approx$1 kbar between experiment and classical force field simulations. *Mol. Phys.* **117** (2019).
